# Maternal Anthropometric Measurements Do Not Have Effect on Birth Weight of Term, Single, and Live Births in Addis Ababa City, Ethiopia

**DOI:** 10.1155/2018/1982134

**Published:** 2018-12-02

**Authors:** Chalachew Bekele Shiferaw, Walelegn Worku Yallew, Gizachew Tadele Tiruneh

**Affiliations:** ^1^St. Paul Hospital Millennium Medical College, Addis Ababa, Ethiopia; ^2^University of Gondar College of Medicine and Health Sciences, Institute of Public Health, Gondar, Ethiopia; ^3^JSI Research and Training Institute Inc./L10K Project, Addis Ababa, Ethiopia

## Abstract

Low birth weight is a global public health problem for mortality and morbidity in any age group. The objective of this study is to investigate the effect of maternal anthropometric measurements on birth weight. A cross-sectional study was conducted from Nov 25, 2012, to Feb 25, 2013, in maternity public hospitals in Addis Ababa city, Ethiopia. The effect is investigated using correlation, linear regression, independent sample T-test, one-way ANOVA, and finally multivariate linear regression analysis. A total of 605 women and their newborns took part in this study and prevalence of low birth weight is 8.3%. On adjusted multivariate linear regression analysis, maternal anthropometric measurements did not have an effect on birth weight. Since maternal mid-upper arm circumference ≤ 20 cm and body mass index ≤18.5kg/m^2^ are almost nil in this study, generalization is difficult to general population where undernourished women are common in the rural Ethiopia and similar study is recommended in those areas. Antenatal care visits, gestational age, and female sex of newborn had statistically significant effect in determining the risk of low birth weight. Women who were living with large family members had a heavier newborn than counterparts. This might be due to the fact that pregnant women have better care and social support in Ethiopian context, so advising pregnant women to live with family members should be considered to enhance social support during pregnancy in Ethiopia. Maternal anthropometric measurements have no significant effect in determining birth weight in the city and we recommend similar studies where undernourished women are common.

## 1. Introduction

Low birth weight (LBW) is a weight at birth less than 2,500 grams irrespective of gestational age. A baby's low weight at birth is either the result of preterm birth (i.e., before 37 weeks of gestation) or the result of restricted fetal (intrauterine) growth [1]. This condition is a worldwide problem, especially where starchy tubers and cereals form the staple food. Africans' major nutritional problem is protein-energy malnutrition; as a result of this, there is a higher percentage of women with low weight, height, and mid-upper arm circumference (MUAC) in the region [2].

Globally, more than 20 million (15%) infants are born with LBW and it is concentrated in two regions of the developing world: Asia and Africa. Seventy-two percent of LBW infants in developing countries are born in Asia and 22% in Africa, of which 13% to 15% found in Sub-Saharan Africa with little variation across the region as a whole [1]. In Ethiopia, the incidence of LBW has significant variation across the area of residence, 17% in rural and 9% of the urban area [3]. Studies in other areas of the country like in Gondar referral teaching hospital and rural population of Kersa also reported 17% and 28% prevalence, respectively [4, 5]. A similar study from Sri Lanka showed that 8.7% of 563 births had LBW [6].

The causes of LBW are multifactorial including complication during pregnancy, genetic, environmental, social-cultural, demographic, and nutritional variables [7, 8]. Women who previously had a child, are educated, and had higher wealth were less likely to deliver a low birth weight baby [9, 10]. Newborns of women who gained more than 24 kg during pregnancy were 148.9g heavier at birth compared to infants of women who gained 8–10 kg and the odds ratio of giving birth to an infant greater than 4000 gram was 2.26 [11]. Overweight and obese women had similar risks of LBW like normal-weight mothers (BMI 18.5-25 kg/m^2^) [12]. The risk of preterm birth appeared significantly higher in overweight and obese women [13]. Wealth status, MUAC, and antenatal care (ANC) were determinants for low birth weight in Kersa, Ethiopia [5]. Prenatal physical activity, depressive symptoms, and social class did not significantly predict offspring weight outcomes [9, 14] whereas in another study depressive and anxiety symptoms were significantly associated with LBW [15].

LBW is closely associated with fetal and neonatal mortality and morbidity, inhibited growth, and cognitive development. It is also a risk factor for chronic diseases later in life like adult-onset diabetes, coronary heart disease, high blood pressure, and intellectual, physical, and sensory disabilities [16].

Anthropometry provides a simple, reliable, and low-cost method of assessing nutritional status by measuring maternal physical structure which can be universally applied at primary care level. Identification of the effect of those anthropometric parameters on birth weight is important in order to determine the level of care and priorities for a referral to centers where reasonable neonatal care is available. As such, the objective of this study is to investigate this to provide evidence-based information that can be used in the future to reduce birth weight-related complications.

## 2. Methods

A cross-sectional study was conducted, in maternity public hospitals of Addis Ababa city, Ethiopia, and we used simple cluster sampling method. First the study participants were divided into four clusters: Tirunesh Beijing, Yekatit-12, Gahandi Memorial, and Zewditu Maternity Hospitals in the city. Yekatit-12 and Gahandi Memorial Maternity Hospitals were selected randomly and then consecutive sampling technique (including all eligible participants) was employed till the calculated sample was achieved in the three-month data collection. Hospitals average monthly client load was 100 and 175 deliveries/month for Yekatit-12 and Gahandi Memorial Hospitals, respectively. Based on this, samples were proportionally allocated 149 for Yekatit-12 Hospital and 456 for Gahandi Memorial Hospital and we finished data collection within three months.

The sample size of 605 was calculated using Open Epi, Version 2, and single proportion formula n= Z(*α*/2)2 P(1-P) /d2 assuming a 17.1% proportion of LBW from previous study in Gondar Teaching Hospital, Ethiopia, at a 95% confidence level and 3% margin of error.

The weight of the newborn, maternal height, mid-upper arm circumference (MUAC), and maternal age were exclusively taken during data collection by the study team. Weight gain during pregnancy, gestational age, date of birth, and history of diabetes mellitus during pregnancy were taken from medical record. Some women did not have antenatal care follow-up and some medical records were not filled correctly; in these cases data was taken by interviewing women by trained midwives within 72 hours of birth. Birth weight was taken using baby weight scales after checking the scale with known weight control. Adult MUAC measuring tape was used to measure maternal MUAC. The height of women was measured on barefoot in centimeter using standard height measuring board and recorded to the nearest 1 cm. Gestational age was taken in weeks starting from last menstrual period (LMP). In cases when women did not know their LMP, health provider's decision on medical records was taken.

The objective of the study was just to see the effect of maternal anthropometrics measurement on birth weight and we tried to see the effect independent of potential factors that affect birth weight. Therefore, maternal diabetes, preterm, postterm, and unknown gestational age were exclusion criteria to reduce potential confounders and stillbirths also were excluded since we could not find outcome variable birth weight for still births.

Data were entered, cleaned, and edited using Epi Info version 3.5.1 and then exported to SPSS version 15 for further analysis. After checking for the fulfillment of the assumptions needed to make inferences about the correlation coefficient, bivariate analysis was done using correlation, independent sample T-test, one-way ANOVA, and simple linear regression. Pearson correlation coefficient (r), unadjusted coefficient B, mean difference, and p-values less than 0.05 were used to fit into the multivariate linear regression model. All independent variables which had the association in bivariate analysis with the p-value of less than 0.05 were included in multiple leaner regression model to determine the independent predictors of birth weight. The variables with p<0.05 in multivariate linear regression analysis were considered as significant predictors of birth weight.

The study was approved by the ethical review board of University of Gondar (UOG), Addis Continental Institute of Public Health (ACIPH) and City Government of Addis Ababa Health Bureau. All eligible study subjects were verbally invited and informed about the expected outcomes, benefits, and risks to take part in this study. They were informed of their right to refuse participating in the research and withdraw at any time they wished to, without losing any of their rights as a client of the hospital.

## 3. Results

### 3.1. Maternal Demographics, Antenatal Care, and Prepregnancy Characteristics

Maternal gravidity, gestational age, and anthropometrics characteristics are shown in Table 1. The mean age of respondents was 26 ± 5 years. Regarding marital status, 94% (567) were married and 6% (38) were single. About 14% (81) of the respondents were illiterate, 38% (221) attended formal elementary education (1-8 years), and 48% (281) attended secondary and above. Out of 605 newborn babies, 320 (53%) were males and 281 (47%) were females.

### 3.2. Newly Born Characteristics

From a total of 603 newborns, 320 (53%) were males and 281 (47%) were females. The mean weight of the newborn is 3085gm and the prevalence of low birth weight is 8.3%. Independent samples T-test was done to see the association of newborn sex with birth weight. The analysis showed that the mean difference in birth weight between male and female was 91gm (Male > female).

### 3.3. Determinants of LBW

#### 3.3.1. Bivariate Analysis

In the first binary linear regression and correlation model maternal variable parameters, gravidity, gestational age, number of ANC, household family size, and maternal age were significantly associated with birth weight (Table 2).

#### 3.3.2. Multivariate Analysis

Adjusted multivariate linear regression analysis is done for independent variables (maternal anthropometric parameters, maternal age, gestational age, gravidity of women, antenatal care visit, household family size, and sex of the newborn) which have significant association on bivariate analysis (Table 3).

## 4. Discussion

Maternal anthropometric characteristics (height, BMI, MUAC, prepregnancy, and term weight) had no significant effect to determine the risk of low birth weight. In India, short mothers had 2.74- and 9.0-fold greater risk of delivering LBW baby than average and tall mothers, respectively [7]. In Sudanese women also a maternal height of < 156 centimetres had a statistically significant effect on determining the risk of LBW, but BMI had no significant effect [17]. Overweight and obese women had similar risks of LBW like normal-weight mothers [12]. In the rural population of Kersa, Ethiopia MUAC is determinant for low birth weight [5]. The possible reason for this difference might be due to lower undernutrition state of women in this study (BMI≤ 20 kg/m^2^ and MUAC ≤ 20 cm were 0.7% and 1%, respectively) and it might be higher in rural population.

Lower family size of women belonged has a significant risk of LBW. Even though the mechanism of action is not clear, this might be due to the following reasons:** First**, when women have a higher family size they could have a higher number of previous pregnancies and older age than their counterparts. Being older and multiparous may determine the birth weight; because women who previously have a child were less likely to deliver a low birth weight (LBW) baby [9, 10] and young maternal age showed a significant risk of low birth weight [8], but both maternal age and parity/gravidity have no effect on birth weight in this study.** Second**, pregnant women who are alone or with some family member might have depression and anxiety symptoms more than pregnant women who are living in large family, because depressive and anxiety symptoms were associated with low birth weight [15].** Third**, social support is widely believed to affect health and feeding practice. Biological fathers and parents coresidence with pregnant mothers had been determinant factors for birth weight in rural South Africa women [18].

Low weight at birth is either the result of preterm birth or the result of restricted fetal (intrauterine) growth [1]. This study is conducted in term birth and the gestational age differences significantly affected birth weight in multivariate linear regression. Antenatal care is determinant for low birth weight [5]. This is because women, who attended antennal care, get the education about feeding, treatment for their illness, supplements, and vaccination. Similarly, in this study lower number of antenatal care visits is an independent determinant factor for LBW.

## 5. Conclusions and Recommendation

Maternal anthropometric measurements have no significant effect in determining birth weight in the city. Since maternal MUAC less than 20 cm and BMI less than 18.5kg/m2 were almost nil in this study, generalization is difficult to sites where undernourished women are common, so we recommended similar study in such areas.

## Figures and Tables

**Table 1:** Maternal age, gravidity, gestational age, and anthropometric characteristics of women at public hospitals in Addis Ababa city, Ethiopia, Nov 25, 2012 to Feb 25, 2013.

***Characteristics***	**N (**%**)**
***Age of mother in years (n=601)***	
≤ 20	77 (12.81%)
21-30	425 (70.70%)
31 – 40	98(16.30%)
≥ 41	01(00.16%)
***Religion (n=600)***	
Orthodox	431(71.83%)
Muslim	115(19.16%)
Protestant	54(09.00%)
***Gravidity of women (n=603)***	
gravid 1	257(42.62%)
gravid 2	175(29.02%)
gravid 3	93(15.42%)
gravid 4	39(06.47%)
gravid 5	19(02.65%)
gravid ≥6	20(3.31.6%)
***Gestational age in weeks (n=605)***	
37 weeks	26(4.30.5%)
38 weeks	130(21.49%)
39 weeks	116(19.17%)
40 weeks	133(21.98%)
41 weeks	116(19.17%)
42 weeks	84(13.88%)
***Mean maternal anthropometric parameters***	***Mean (±SD)***
Maternal height (cm)	156± 06
Pre-pregnancy weight (gm.)	54835 ± 8861
Term weight (gm.)	63765±9205
Pregnancy weight gain (gm.)	9150±5746
Term BMI (kg/m2)	26±03
Pre-pregnancy BMI (kg/m2)	22±03
MUAC (cm)	*26±03*

**Table 2:** Bivariate linear regression analysis between maternal age, anthropometry, family size, number of ANC, gravidity, gestational age, and income with birth weight.

*Independent variables*	*Coefficients*			
	B	Std. error	t	Sig.
MUAC	27.323	6.301	4.337	< 0.001
Height	14.071	3.184	4.419	< 0.001
Pre-pregnancy weight	.013	.002	5.128	< 0.001
Term weight	.014	.002	6.025	<0.001
Pregnancy weight gain	.010	.004	2.452	0.015
Term BMI	28.308	6.354	4.455	< 0.001
Pre-pregnancy BMI	20.290	6.489	3.127	.002
Gravidity of mother	44.494	15.359	2.897	.004
Gestational age in weeks	55.265	13.822	3.998	< 0.001
Number of ANC visits	26.719	7.912	3.377	.001
Household family size women belonged	58.635	14.308	4.098	< 0.001
Maternal age in years	17.909	4.201	4.263	< 0.001

**Table 3:** Multivariate linear regression showing the association between dependent variables and dependent variable.

Model	Unstandardized Coefficients		t	Sig	95% Confidence Interval for B	
Independent variables	B	Std. Error			Lower bound	Upper bound
Constant	-2316.99	1933.22	-1.19	.231	-6116.73	1482.74
MUAC of women	-8.95	10.48	-.85	.394	-29.56	11.66
Pre-pregnancy weight	.000	.008	-.06	.950	-.016	.015
Height of women	12.13	11.50	1.05	.292	-10.47	34.73
Term weight of women	.001	.013	.08	.936	-.02	.027
Term BMI of women	39.75	32.28	1.23	.219	-23.69	103.20
Pre-pregnancy BMI of women	-9.85	20.07	-.49	.624	-49.30	29.59
**Sex of the newborn**	**-128.84**	**44.32**	**-2.90**	**.004**	**-215.95**	**-41.73**
Gravidity of women	-16.92	23.77	-.71	.477	-63.65	29.80
**Gestational age in weeks**	**64.09**	**15.50**	**4.13**	**<0.001**	**33.62**	**94.55**
**Number of ANC visits**	**22.86**	**9.20**	**2.48**	**.013**	**4.77**	**40.94**
**HH family size **	**59.07**	**18.77**	**3.14**	**.002**	**22.18**	**95.96**
Maternal age	9.05	5.60	1.61	.107	-1.96	20.07

## Data Availability

The data used to support the findings of this study are included within the article.
